# miR-410 induces both epithelial–mesenchymal transition and radioresistance through activation of the PI3K/mTOR pathway in non-small cell lung cancer

**DOI:** 10.1038/s41392-020-0182-2

**Published:** 2020-06-12

**Authors:** Yue Yuan, Hu Liao, Qiang Pu, Xixian Ke, Xueting Hu, Yongfang Ma, Xinmei Luo, Qianqian Jiang, Yi Gong, Min Wu, Lunxu Liu, Wen Zhu

**Affiliations:** 1 State Key Laboratory of Biotherapy and Cancer Center, West China Hospital, Sichuan University and Collaborative Innovation Center of Biotherapy, Chengdu, Sichuan China; 2 Department of Thoracic Surgery, West China Hospital, Sichuan University, Chengdu, Sichuan China; 3 Department of Biomedical Sciences, University of North Dakota, Grand Forks, North Dakota USA

**Keywords:** Lung cancer, Non-coding RNAs

## Abstract

Radiotherapy remains one of the major treatments for non-small cell lung cancer (NSCLC) patients; whereas intrinsic or acquired radioresistance limits its efficacy. Nevertheless, most studies so far have only focused on acquired resistance. The exact mechanisms of intrinsic radioresistance in NSCLC are still unclear. A few studies have suggested that epithelial–mesenchymal transition (EMT) is associated with radioresistance in NSCLC. However, little is known about whether the abnormal expression of specific microRNAs induces both EMT and radioresistance. We previously found that miR-410 has multiple roles as an oncomiRNA in NSCLC. In this study, we revealed that miR-410 overexpression promoted EMT and radioresistance, accompanied by enhanced DNA damage repair both in vitro and in vivo. Conversely, knockdown of miR-410 showed the opposite effects. We further demonstrated that PTEN was a direct target of miR-410 by using bioinformatic tools and dual-luciferase reporter assays, and the miR-410-induced EMT and radioresistance were reversed by PI3K, Akt, and mTOR inhibitors or by restoring the expression of PTEN in NSCLC cells. In addition, we preliminarily found that the expression of miR-410 was positively correlated with EMT and negatively associated with the expression of PTEN in NSCLC specimens. In summary, these results demonstrated that miR-410 is an important regulator on enhancing both NSCLC EMT and radioresistance by targeting the PTEN/PI3K/mTOR axis. The findings suggest that miR-410-induced EMT might significantly contribute to the enhanced radioresistance. Therefore, miR-410 may serve as a potential biomarker or therapeutic target for NSCLC radiotherapy.

## Introduction

Radiotherapy (RT), stereotactic body radiation therapy (SBRT) in particular, is a standard treatment for early-stage non-small cell lung cancer (NSCLC) patients who are unfit for surgery.^1,2^ In addition, many patients diagnosed with NSCLC are already at an advanced stage,^3^ and RT/chemotherapy is recommended as radical or palliative treatment.^4^ However, similar to other epithelial tumor cells, NSCLC cells are often resistant to radiation.^5^ The intrinsic radioresistance of lung cancer cells leads to treatment failure in numerous NSCLC patients at the first RT. In addition, lung cancer cells may acquire radioresistance after primary RT;^6^ therefore, most patients show local recurrence after RT.^7^ Hence, intrinsic or acquired resistance to RT remains a major obstacle that limits the efficiency of the treatment for NSCLC.^8,9^ Nevertheless, most studies have focused on acquired resistance, and the exact mechanisms of intrinsic radioresistance in NSCLC are still unclear, likely owing to tumor heterogeneity and the various factors involved. A better understanding of the underlying mechanisms of intrinsic radioresistance may help to discover novel biomarkers or targets for improving the radioresponse of NSCLC.

Recently, a few studies have demonstrated that epithelial–mesenchymal transition (EMT) is positively associated with radioresistance in NSCLC.^10–12^ However, most of these studies have mainly focused on radiation-induced changes in EMT characteristics. It remains unclear whether the occurrence of EMT can result in radioresistance in NSCLC, and little is known about whether the abnormal expression of genes or pathways can affect both EMT and radiosensitivity in NSCLC. Notably, accumulating evidence has confirmed that EMT has a vital role in the regulation of NSCLC malignancy and is also of prognostic relevance.^13^ Therefore, exploring the molecular mechanisms underlying the regulation of both EMT and radioresistance will lay the basis for understanding whether EMT directly causes radioresistance. It is also important to discover novel mechanisms of radioresistance in NSCLC.

microRNAs (miRNAs) are important regulators in diverse biological processes of cancers, including tumor radiosensitivity, and they may serve as radiosensitizers or potential biomarkers in RT.^14,15^ Recently, miR-875-5p was determined to increase radiosensitivity and counteract EMT in prostate cancer.^16^ In NSCLC, it has been demonstrated that specific miRNAs are involved in the regulation of radioresistance or the EMT process.^17,18^ Recently, the only study suggested that miR-148b might affect the radioresponse and EMT in A549 cells.^19^ However, researches on the radioresistance of miRNAs in NSCLC are still limited. High-quality, in-depth mechanistic studies are still needed to elucidate the role and potential mechanisms of specific miRNAs in regulating the NSCLC radioresponse.^20^ Moreover, research on how miRNAs mediate the association between EMT and radioresistance in NSCLC is still largely in its infancy, and further studies are needed to discover more about the underlying mechanisms.^21^ It also remains unclear whether the abnormal expression of miRNAs can regulate both EMT and radiosensitivity in NSCLC.

miR-410 is a member of the miR-379/410 miRNA cluster, which has crucial roles in various biological processes and cancer malignancies.^22,23^ In our previous studies,^24,25^ the upregulation of miR-410 was identified in NSCLC cells, including A549 and H1299 cells, and in NSCLC specimens. Moreover, overexpression of miR-410 was found to promote cell proliferation, metastasis, stemness, and chemoresistance by directly targeting SLC34A2 or Gsk3β and activating the Wnt/β-catenin pathway. Our previous studies demonstrated for the first time the role of miR-410 as an oncogene in tumorigenesis and the development of NSCLC. Likewise, miR-410 was found to increase the levels of phosphorylated Akt in NSCLC.^26^ Interestingly, A549 and H1299 cells with wild-type EGFR exhibited resistance to radiation and were used as radioresistant cells.^11,27,28^ Stabilization or upregulation of Slug by the inhibition of Gsk3β or activation of the β-catenin pathway could result in EMT and thus increase metastasis and chemoresistance in NSCLC.^29,30^ In addition, cancer stem cells (CSCs) and Akt phosphorylation are positively correlated with radioresistance and EMT.^13,31,32^ Consequently, mounting evidence indicates that miR-410 might be a promoter of EMT and radioresistance in NSCLC.

In the present study, we revealed that miR-410 induced both NSCLC EMT and radioresistance by targeting the PTEN/PI3K/mTOR axis in vitro and in vivo, and the promotion of radioresistance might be associated with enhanced DNA damage repair. Moreover, the data also suggested that miR-410-induced EMT might significantly contribute to the enhanced radioresistance, which might be a novel mechanism of radioresistance in NSCLC. Further investigation with NSCLC specimens also demonstrated that miR-410 was positively correlated with EMT. Collectively, our results indicate that miR-410 may be a potential biomarker or therapeutic target in NSCLC RT.

## Results

### miR-410 promoted EMT and radioresistance in NSCLC cell lines

**Fig. 1:**
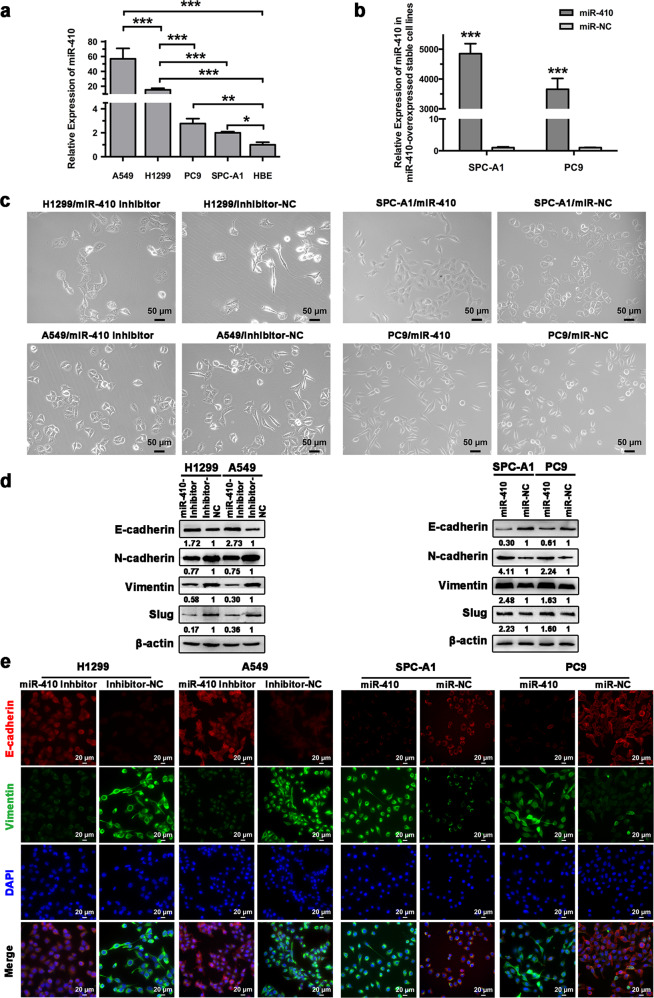
miR-410 promoted EMT in NSCLC cell lines. **a** qRT-PCR analysis to quantify the endogenous levels of miR-410 in NSCLC cell lines (A549, H1299, PC9, and SPC-A1) and a non-cancerous bronchial epithelial cell line (HBE). U6 was used as a control. Experiments were performed in triplicate, and the relative expression levels were displayed as the mean ± SD. ^*^P < 0.05; ^**^P < 0.01; ^***^P < 0.001. **b** qRT-PCR analysis of miR-410 levels in the indicated cell lines. (miR-410 and miR-NC, miR-410-overexpressing stable cell line and its matched NC control stable cell line). Experiments were performed in triplicate, and the relative expression levels were displayed as the mean ± SD. ^*^P < 0.05; ^**^P < 0.01; ^***^P < 0.001. **c** Representative morphological images of the indicated NSCLC cells. Scale bar: 50 µm. **d** Western blotting analysis of EMT markers (E-cadherin, N-cadherin, Vimentin, and Slug) in the indicated cells. β-Actin was used as an internal control. **e** Immunofluorescence images of EMT markers (E-cadherin and vimentin) in the indicated cells. Scale bar: 20 µm

**Fig. 2:**
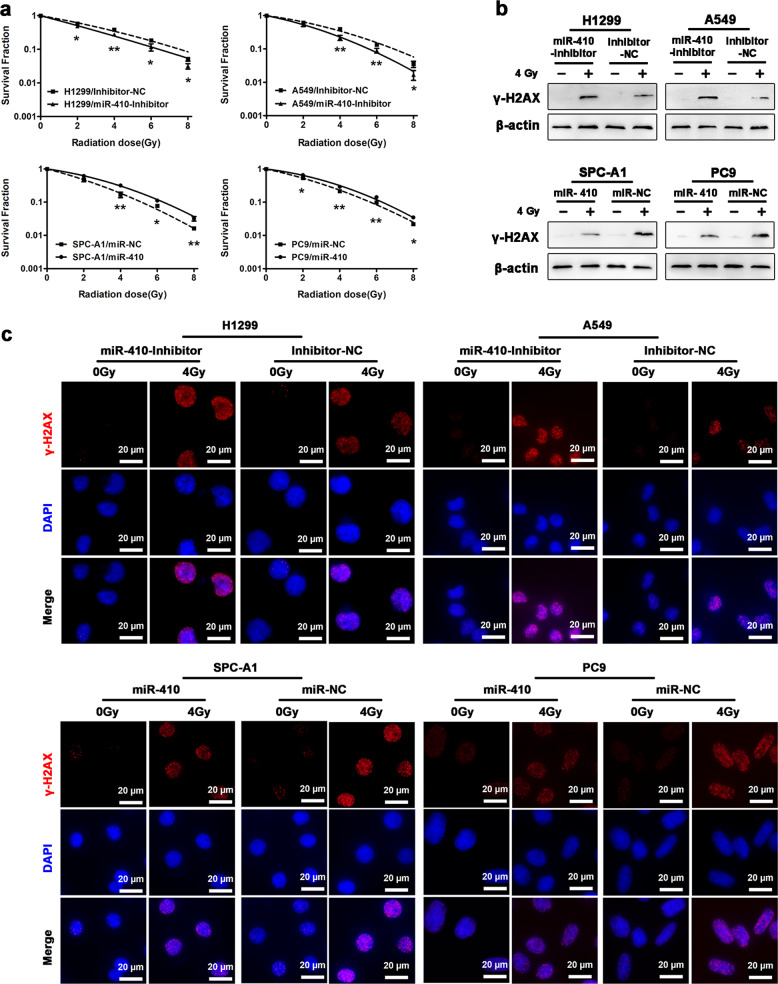
miR-410 increased NSCLC cell radioresistance. **a** Clonogenic survival assays of the radioresponse in the indicated NSCLC cells. Dose–survival curves were created by survival fractions. Experiments were performed in triplicate, and the values were presented as the mean ± SD. ^*^P < 0.05; ^**^P < 0.01. **b** Western blotting analysis of γ-H2AX in the indicated cells after 4 h of 4 Gy radiation. **c** Representative immunofluorescence images of nuclear γ-H2AX foci (cell nuclei: blue; γ-H2AX foci: red) in the indicated cells after 4 h of 4 Gy radiation. Scale bar: 20 µm

To understand the role of miR-410 in EMT and radioresponse in NSCLC cells, the endogenous levels of miR-410 in NSCLC cell lines (A549, H1299, PC9, and SPC-A1) and a non-cancerous bronchial epithelial cell line (HBE) were assessed first. The miR-410 expression levels were significantly higher in NSCLC cells than in HBE cells and were higher in A549 and H1299 cells than in PC9 and SPC-A1 cells (Fig. 1a). Thus, stable miR-410-overexpressing PC9 and SPC-A1 cell lines were established (termed as PC9-miR-410 and SPC-A1-miR-410) (Fig. 1b), and the previously established stable miR-410 knockdown A549 and H1299 cell lines (termed as A549-Inh and H1299-Inh) were also used to further explore the effects of miR-410 on EMT and the radioresponse in vitro. Interestingly, morphological changes from the typical spindle-like shape of A549 and H1299 cells to a cobblestone-like shape were observed upon miR-410 knockdown, whereas ectopic expression of miR-410 resulted in more PC9 and SPC-A1 cells with a spindle-like shape (Fig. 1c). In addition, the protein levels of epithelial marker (E-cadherin) were notably increased, and mesenchymal markers (N-cadherin, Vimentin, and Slug) were significantly decreased in A549-Inh and H1299-Inh cells. Conversely, miR-410 overexpression caused the opposite effects (Fig. 1d). Similar results were shown by immunofluorescence assay of E-cadherin and vimentin (Fig. 1e, Supplemental Fig. S1). Moreover, it has been demonstrated in our previous studies that overexpression of miR-410 promotes cell invasion and migration.^24,25^ Taken together, the data demonstrated that overexpression of miR-410 could promote the EMT process in NSCLC cells. To further assess the effect of miR-410 on the radioresponse in vitro, cell radiosensitivity was determined by clonogenic survival assays. Our results showed that radioresistance was significantly decreased in A549-Inh and H1299-Inh cells but significantly increased in PC9-miR-410 and SPC-A1-miR-410 cells (Fig. 2a). Then, we further investigated the effect of miR-410 on irradiation-induced DNA double-strand breaks (DSBs) by detecting the expression of γ-H2AX, a specific marker of DSBs.^33^ The protein levels of γ-H2AX at 4 h after irradiation were significantly higher in A549-Inh and H1299-Inh cells but significantly lower in PC9-miR-410 and SPC-A1-miR-410 cells (Fig. 2b). Moreover, the γ-H2AX foci detected by immunofluorescence showed similar results in NSCLC cells (Fig. 2c). Thus, the data revealed that miR-410 increased radioresistance and might be associated with enhanced DSB repair in NSCLC cells.

### miR-410 directly targeted PTEN in NSCLC cells

**Fig. 3:**
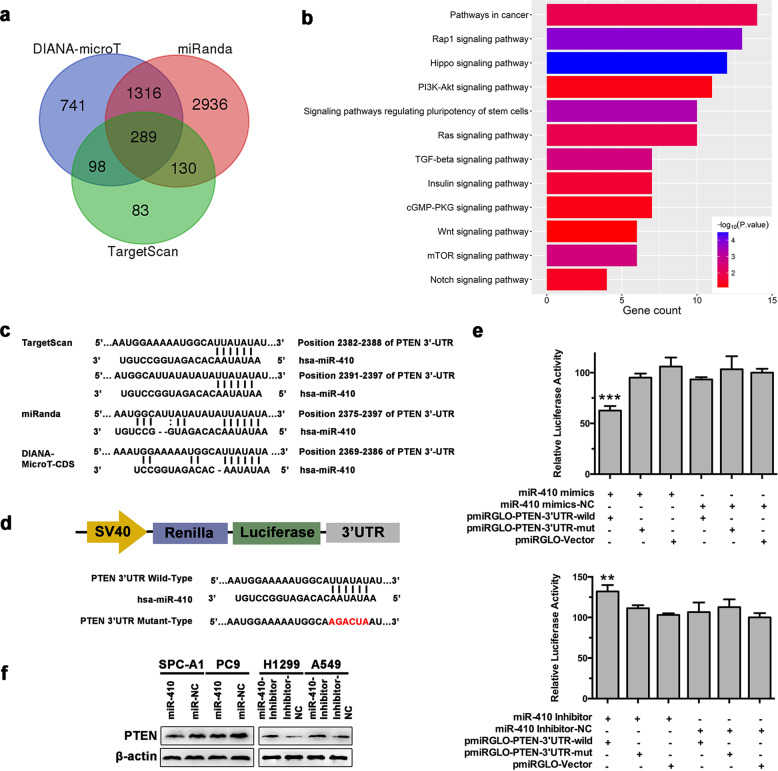
miR-410 directly targeted PTEN in NSCLC cells. **a** Venn diagrams of the number of genes identified as potential targets of miR-410 by three algorithms: TargetScan, miRanda, and DIANA-microT. **b** The KEGG analysis of potential miR-410 targets (*n* = 289) by DAVID bioinformatics resources. **c** Binding sites of PTEN 3′-UTR sequences with miR-410 seed sequences predicted by the algorithms TargetScan, miRanda, and DIANA-microT. **d** Sequence alignment between miR-410 sequences and PTEN 3′-UTR wild-type or PTEN 3′-UTR mutant sequences. Red indicates the sequences of the mutated miR-410 binding sites. **e** Relative luciferase activity of luciferase reporters containing wild-type or mutant PTEN 3′-UTR co-transfected with miR-410 mimics or inhibitors or negative control in 293T cells. Experiments were performed in triplicate, and the values were presented as the mean ± SD. ^*^P < 0.05; ^**^P < 0.01; ^***^P < 0.001. **f** The protein levels of PTEN in the indicated NSCLC cells

To identify the mechanism underlying the miR-410-induced EMT process and radioresistance, TargetScan, DIANA-microT-CDS, and miRanda were used to predict the targets of miR-410 (Fig. 3a). The potential targets predicted by all three algorithms (289 genes) were submitted for KEGG pathway analysis conducted with DAVID tools thereafter. The results showed that PTEN was among the 289 potential targets. Moreover, previous studies have showed that PTEN is closely related to EMT and radioresistance.^34,35^ In addition, KEGG analysis suggested that the predicted targets were involved in several cancer-related pathways (Fig. 3b, Supplemental Fig. S2). Among them, the PI3K/Akt and mTOR signaling pathways have been demonstrated to be closely associated with PTEN,^36^ which further indicated that PTEN might be a candidate target of miR-410. The direct interaction between miR-410 and the 3′-UTR of PTEN was then confirmed by a dual-luciferase reporter assay (Fig. 3c–e). Moreover, elevated protein levels of PTEN were observed in A549-Inh and H1299-Inh cells, whereas reduced expression of PTEN was detected in PC9-miR-410 and SPC-A1-miR-410 cells (Fig. 3f). However, the mRNA levels of PTEN showed no difference with the change in miR-410 expression (Supplemental Fig. S3). Collectively, these results confirmed that PTEN was a direct target of miR-410 and that it was posttranscriptionally regulated by miR-410 in NSCLC cells.

### The PI3K/mTOR pathway contributed to miR-410-induced EMT and radioresistance in NSCLC cells

**Fig. 4:**
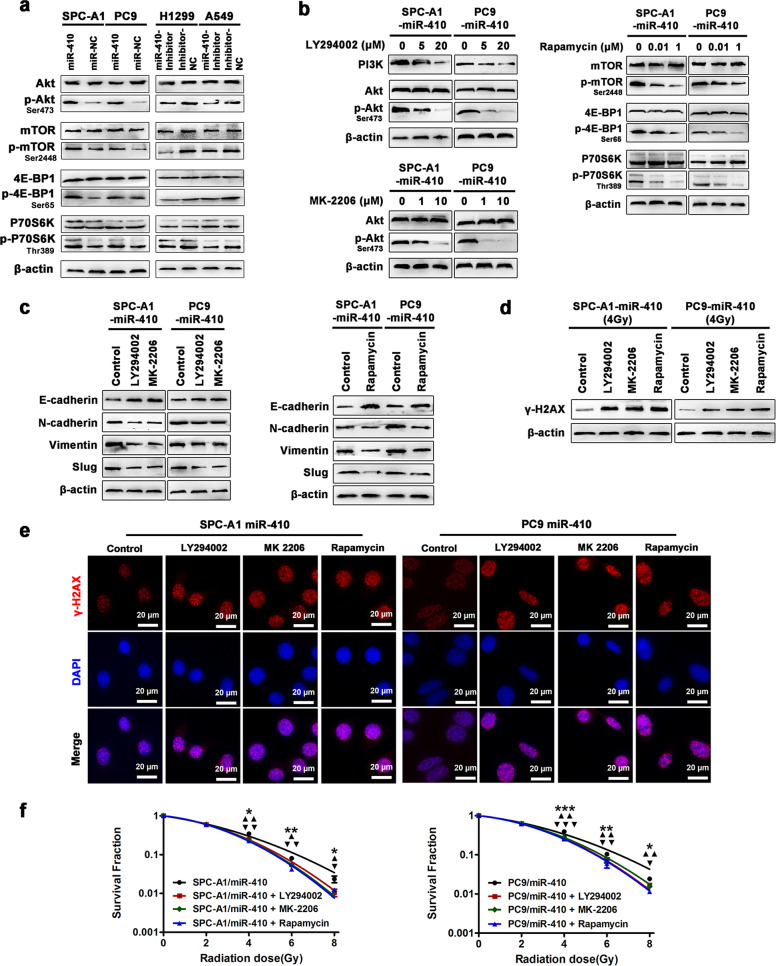
miR-410 promoted EMT and radioresistance via activation of the PI3K/mTOR pathway in NSCLC cells. **a** Western blotting analysis of total Akt, phospho-Akt, total mTOR, phospho-mTOR, total 4E-BP1, phospho-4E-BP1, total P70S6K, and phospho-P70S6K in the indicated cells. **b** Western blotting analysis of PI3K/mTOR pathway markers in the indicated cells treated with LY294002, MK-2206, or rapamycin at various concentrations. **c** Western blotting analysis of EMT markers in the indicated cells treated with LY294002 (20 μm), MK-2206 (10 μm), or rapamycin (1 μm). **d** Western blotting analysis of γ-H2AX in the indicated cells treated with LY294002 (20 μm), MK-2206 (10 μm), or rapamycin (1 μm) after exposure to 4 Gy of irradiation. **e** Representative immunofluorescence images of nuclear γ-H2AX foci (cell nuclei: blue; γ-H2AX foci: red) in the indicated cells treated with LY294002 (20 μm), MK-2206 (10 μm), or rapamycin (1 μm) after 4 h of 4 Gy radiation. Scale bar: 20 µm. **f** Clonogenic survival assays of the radioresponses in the indicated cells treated with LY294002 (20 μm), MK-2206 (10 μm), or rapamycin (1 μm). Experiments were performed in triplicate, and the values were presented as the mean ± SD. ^*^/^▲^/^▼^, *P* < 0.05; ^**^/^▲▲^/^▼▼^, *P* < 0.01; ^***^/^▲▲▲^/^▼▼▼^, *P* < 0.001 *, treated with LY294002; ^▲^, treated with MK-2206; ^▼^, treated with rapamycin

Studies have demonstrated that the activation of the PI3K/mTOR signaling pathway plays vital roles in regulating the EMT process or radiosensitivity in NSCLC.^37,38^ Therefore, based on the results above, we tested whether miR-410 might exert its biological functions by activating the PI3K/mTOR pathway. Of note, miR-410 overexpression in PC9 and SPC-A1 cells robustly increased the levels of phosphorylated Akt and mTOR, resulting in the enhanced phosphorylation of P70S6K and 4E-BP1. In contrast, miR-410 knockdown in A549 and H1299 cells remarkably decreased the levels of phosphorylated Akt, mTOR, P70S6K, and 4E-BP1 (Fig. 4a). These results suggested that miR-410 indeed activated the PI3K/mTOR pathway in NSCLC cells. Based on the results above that overexpression of miR-410 could promote both EMT and radioresistance in vitro, we next investigated the effects of specific blockade of the PI3K, Akt, or mTOR pathway on miR-410-induced EMT and radioresistance in miR-410-overexpressing PC9 and SPC-A1 cells. The effectiveness of different doses of PI3K inhibitor (LY294002), Akt inhibitor (MK-2206), and mTOR inhibitor (rapamycin) was confirmed first (Fig. 4b). Further results showed that treatment with the specific PI3K, Akt or mTOR inhibitor drastically decreased the expression of N-cadherin, Vimentin, and Slug while elevating the expression of E-cadherin in PC9-miR-410 and SPC-A1-miR-410 cells (Fig. 4c). In addition, the protein levels and nuclear foci of γ-H2AX in PC9-miR-410 and SPC-A1-miR-410 cells were considerably increased after irradiation with the inhibition of the PI3K, Akt, or mTOR pathway (Fig. 4d, e). Similarly, the radioresistance of PC9-miR-410 and SPC-A1-miR-410 cells was significantly decreased after treatment with the PI3K, Akt, or mTOR inhibitor (Fig. 4f). Hence, these data illustrated that miR-410 promoted both the EMT process and radioresistance in NSCLC cells by activating PI3K/mTOR signaling.

### PTEN was essential for miR-410-induced EMT and radioresistance in NSCLC cells

**Fig. 5:**
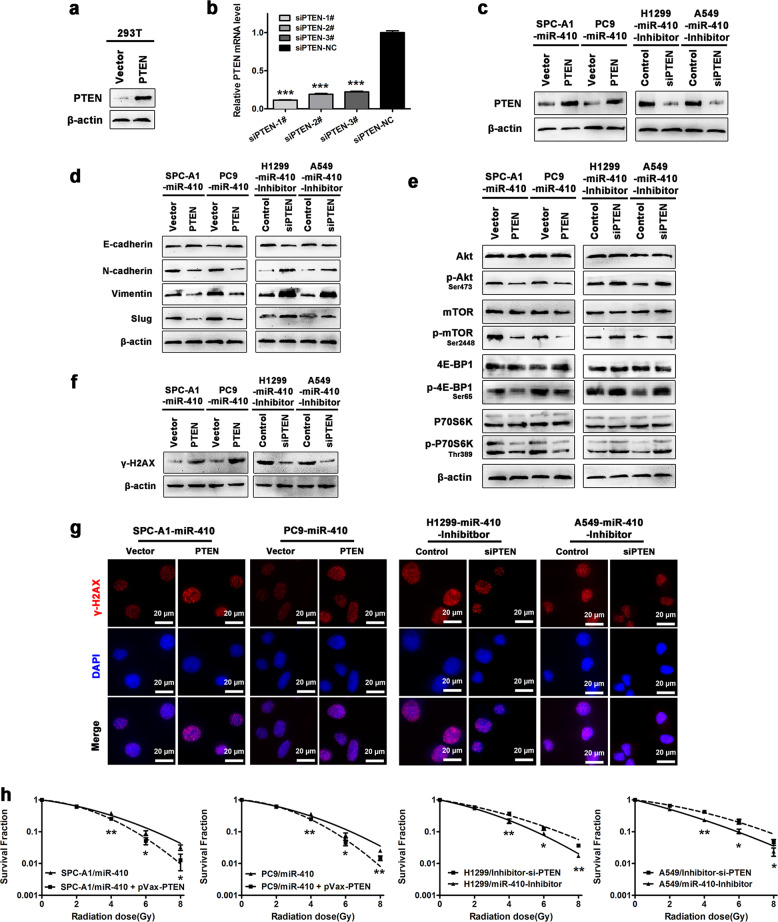
Restoration of PTEN reversed miR-410-induced EMT and radioresistance in NSCLC cells. **a** Western blotting analysis of PTEN protein levels in 293T cells after transfection with pVax-PTEN/pVax. **b** qRT-PCR analysis of PTEN mRNA levels in 293T cells after transfection with #1, #2, #3-PTEN siRNA (siPTEN), or siRNA-NC (siPTEN-NC). Experiments were performed in triplicate, and the relative expression levels were displayed as the mean ± SD. ^*^P < 0.05; ^**^P < 0.01^; ***^P < 0.001. **c** Western blotting confirmation of the restoration of PTEN and the depletion of PTEN in indicated cells. **d** Western blotting analysis of EMT markers after restoration of PTEN and depletion of PTEN in the indicated cells. **e** Western blotting analysis of PI3K/mTOR pathway markers after restoration of PTEN and depletion of PTEN in the indicated cells. **f** Western blotting analysis of γ-H2AX after restoration of PTEN and depletion of PTEN in the indicated cells with exposure to 4 Gy of irradiation. **g** Representative immunofluorescence images of nuclear γ-H2AX foci (cell nuclei: blue; γ-H2AX foci: red) after restoration of PTEN and depletion of PTEN in the indicated cells after 4 h of 4 Gy radiation. **h** Clonogenic survival assays of the radioresponse in the indicated cells after restoration of PTEN and depletion of PTEN. Experiments were performed in triplicate, and the values were presented as the mean ± SD. ^*^P < 0.05; ^**^P < 0.01; ^***^P < 0.001

We further investigated whether PTEN is essential for PI3K/mTOR activation, thus resulting in the biological functions of miR-410 in NSCLC cells. The protein levels of PTEN were remarkably increased after transfecting 293T cells with a PTEN expression plasmid (pVax-PTEN) (Fig. 5a). Moreover, #1 siPTEN exhibited the most significant reduction in PTEN mRNA in 293T cells and was thus chosen for the following study (Fig. 5b). In addition, the effectiveness of pVax-PTEN and siPTEN was further confirmed in miR-410 overexpression or knockdown NSCLC cells, respectively (Fig. 5c). Indeed, restoration of PTEN expression in PC9-miR-410 and SPC-A1-miR-410 cells significantly inhibited the expression of N-cadherin, Vimentin and Slug and increased the expression of E-cadherin (Fig. 5d). Moreover, restoration of PTEN expression drastically decreased the levels of phosphorylated Akt, mTOR, P70S6K, and 4E-BP1 in PC9-miR-410 and SPC-A1-miR-410 cells (Fig. 5e). In addition, the expression levels of γ-H2AX and the radiosensitivity of PC9-miR-410 and SPC-A1-miR-410 cells were greatly increased after irradiation with the restoration of PTEN (Fig. 5f–h). Conversely, transfecting siPTEN in A549-Inh and H1299-Inh cells resulted in the opposite effects (Fig. 5d–g). In conclusion, these data demonstrated that PTEN was essential for miR-410-induced activation of the PI3K/mTOR pathway, thus resulting in enhanced EMT and radioresistance in NSCLC cells.

### miR-410 promoted EMT and radioresistance in vivo and was associated with targeting PTEN while activating the PI3K/mTOR pathway

**Fig. 6:**
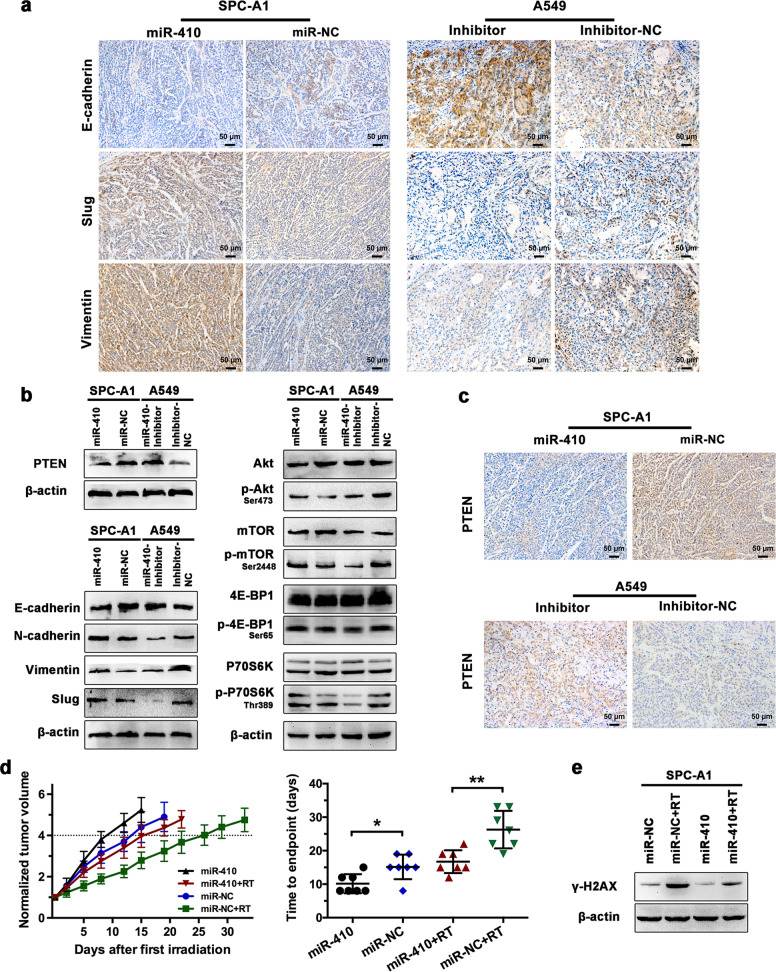
Overexpressing miR-410 promoted EMT and radioresistance in vivo and was associated with the PTEN/PI3K/mTOR axis. **a** Immunohistochemical (IHC) staining of EMT markers in tumors recovered from mice transplanted with the indicated cell lines. Scale bars, 50 μm. **b** Western blotting analysis of PTEN, EMT markers, and PI3K/mTOR pathway markers in the indicated tumor tissues. **c** IHC staining of PTEN in the indicated tumor tissues. Scale bars, 50 μm. **d** The means of tumor volumes normalized to the original volumes in the indicated groups. Dotted line, the endpoint of four times the original volume. Tumor volume quadrupling time of the indicated groups. SPC-A1 miR-410 and miR-NC tumors were treated with a weekly 10 Gy dose of radiotherapy (RT) for 3 weeks or mock RT (0 Gy). The values were shown as the means ± SD (*n* = 7/group). ^*^P < 0.05; ^**^P < 0.01; ^***^P < 0.001. **e** Western blotting analysis of γ-H2AX in the indicated tumor tissues

To determine whether miR-410 overexpression could promote EMT in vivo, SPC-A1-miR-410 cells were injected into the flanks of mice, and the A549-Inh subcutaneous tumor model established previously was also used.^25^ Consistent with the results in vitro, immunohistochemical staining of Vimentin and Slug was remarkably increased, whereas E-cadherin was significantly decreased in SPC-A1-miR-410 tumors (Fig. 6a). Accordingly, the protein levels of E-cadherin, N-cadherin, Vimentin, and Slug showed similar results. Moreover, elevated expression levels of phosphorylated Akt, mTOR, P70S6K, and 4E-BP1 were also observed in SPC-A1-miR-410 tumor tissues (Fig. 6b). Notably, both the protein levels and immunohistochemical staining of PTEN in the SPC-A1-miR-410 group exhibited significant downregulation (Fig. 6b, c). Conversely, investigations of A549-Inh tumors showed the opposite results (Fig. 6a–c). Thus, these data suggested that miR-410 overexpression could enhance the EMT process in vivo and was associated with targeting PTEN while activating the PI3K/mTOR pathway.

Moreover, to explore the effects of miR-410 overexpression on radioresponse in vivo, mice with subcutaneous tumors generated by SPC-A1-miR-410 and SPC-A1-miR-NC cells were randomly administered RT or mock RT. The SPC-A1-miR-410 tumors were considerably more radioresistant, as displayed by a shorter growth delay than the SPC-A1-miR-NC tumors (6.57 days (miR-410) vs. 11.14 days (miR-NC)) (Fig. 6d, Supplemental Table S1). In addition, the protein levels of γ-H2AX were greatly decreased in SPC-A1-miR-410 tumors treated with irradiation compared with SPC-A1-miR-NC tumors treated with irradiation (Fig. 6e). Altogether, these data demonstrated that miR-410 overexpression promoted radioresistance accompanied by enhanced DSB repair in vivo, and the enhanced radioresistance in vivo was also related to the activation of the PTEN/PI3K/mTOR axis.

### miR-410 overexpression was correlated with EMT and low expression of PTEN in NSCLC specimens

**Fig. 7:**
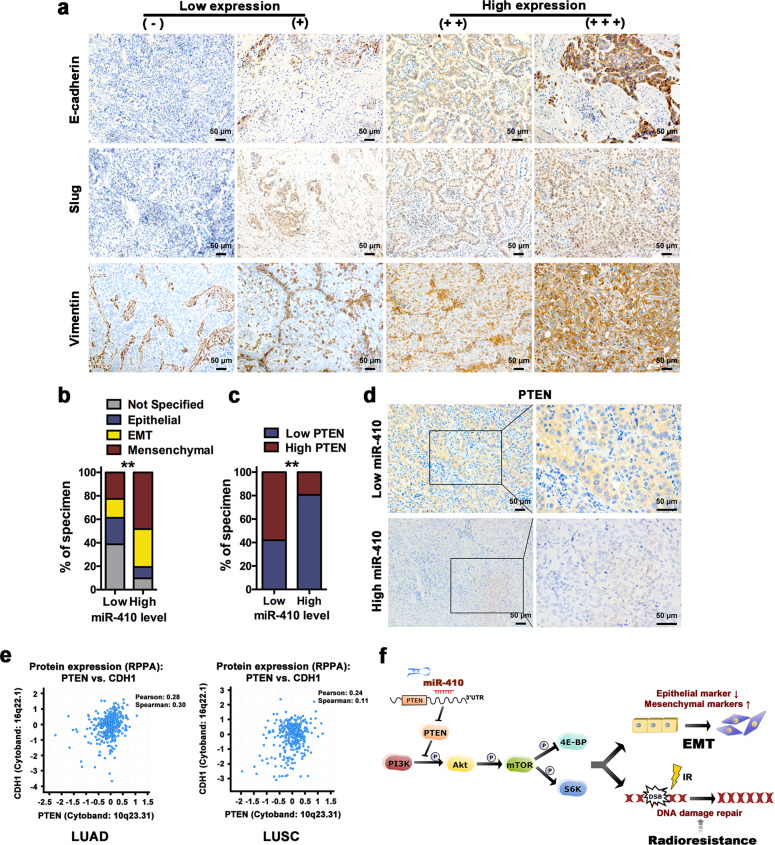
miR-410 overexpression correlated with EMT and low expression of PTEN in NSCLC tumor tissues. **a** IHC staining yielded grades ranging from (−) to (+++), which were representative of the expression of EMT markers in NSCLC specimens. A grading of (−) and (+) represented low expression of EMT markers, and (++) to (+++) represented high expression. Scale bars, 50 μm. **b** Percentage of specimens displaying EMT phenotype status in patient specimens, respectively with low and high miR-410 expression. **c** Percentage of specimens showing low or high expression of PTEN in patient samples, respectively, with low and high miR-410 expression. **d** Representative IHC images of PTEN expression in cases with low and high miR-410. Scale bars, 50 μm. **e** Correlations of the protein levels of PTEN with E-cadherin (CDH1) in 360 cases of lung adenocarcinoma (TCGA, provisional) and 325 cases of lung squamous carcinoma (TCGA, provisional) from the cBioPortal bioinformatics database. **f** Schematic representation of the proposed molecular mechanism of the miR-410-mediated pathway in NSCLC EMT and radioresistance. ^*^P < 0.05; ^**^P < 0.01; ^***^P < 0.001

In an effort to explore the clinical relevance of miR-410 and the EMT process, 62 NSCLC samples were collected. Then, the expression levels of miR-410 and EMT markers were examined, and the EMT phenotypes were classified based on our defined criteria (Fig. 7a). The high (>median, *n* = 31) and low (<median, *n* = 31) miR-410 expression groups were identified. Consequently, mesenchymal and EMT phenotypes were more likely found in patients with high miR-410 expression (Fig. 7b, Supplemental Table S2). High miR-410 expression was also linked with high expression of Vimentin (Supplemental Table. S3). Taken together, these data suggested a positive correlation between miR-410 and the EMT process in NSCLC tissues.

Moreover, 80.65% of high miR-410 expression specimens showed low expression of PTEN, and 58.06% of low miR-410 expression specimens showed high expression of PTEN (Fig. 7c, d). This result indicated that the inverse correlation between miR-410 and PTEN was also clinically relevant in NSCLC.

Owing to the limited miR-410 expression data in cBioPortal, we explored the correlation between the expression of PTEN and EMT markers. Of note, in 360 cases of lung adenocarcinoma (TCGA, provisional) and 325 cases of lung squamous carcinoma (TCGA, provisional), the protein levels of PTEN were positively associated with E-cadherin levels (Fig. 7e). Hence, these data preliminarily further indicated the positive correlation between miR-410 and the EMT process in TCGA NSCLC specimens.

Furthermore, patients with high miR-410 expression tended to show poorer overall survival in 542 cases of lung adenocarcinoma tissues (TCGA_LUAD), whereas no significant difference was observed in 416 cases of lung squamous carcinoma tissues (TCGA_LUSC), which were analyzed by PROGmiRV2 (Supplemental Fig. S4). Thus, the results preliminarily showed that miR-410 overexpression is associated with poor prognosis in lung adenocarcinoma and indicated the role of miR-410 as an oncomiRNA, which was consistent with our previous studies.^25^

## Discussion

It has been reported that miRNAs are involved in the regulation of radiosensitivity and may be predictive biomarkers or therapeutic targets in cancer RT.^15^ However, little is known about whether the abnormal expression of miRNAs promotes both EMT and radioresistance. In the current study, we revealed the roles and the underlying mechanism of miR-410 in inducing both EMT and radioresistance, which also suggested that miR-410-induced EMT might significantly contribute to the enhanced radioresistance.

The occurrence of EMT elicits alterations in cell morphology and the activation of “EMT-inducing transcription factors”, including the Snail family, which results in changes in EMT-associated markers.^39^ Consistent with these findings, in our study, morphological changes into spindle-like shapes were observed in PC9-miR-410 and SPC-A1-miR-410 cells, accompanied by a remarkable reduction in E-cadherin levels and an increase in N-cadherin, Vimentin, and Slug levels. Conversely, the opposite effects were found in A549-Inh and H1299-Inh cells. Hence, our data demonstrated that miR-410 overexpression promoted the EMT process in NSCLC cells.

Only a few studies have shown that EMT is positively correlated with radioresistance in NSCLC. On the one hand, NSCLC cells that survived radiation and grew in spheres showed mesenchymal phenotypes, and inhibition of radiation-induced EMT could enhance the radiosensitivity of cells.^10,11^ Upregulation of mesenchymal markers and reduction of epithelial markers were also observed in NSCLC specimens after chemoradiotherapy.^40^ On the other hand, mesenchymal lung cancer cells were found to display increased radioresistance,^12^ which suggested that EMT might in turn contribute to radioresistance in NSCLC. In our study, assessments of the radioresponse in miR-410 overexpression or knockdown cells demonstrated that miR-410 could increase radioresistance, as well as promote EMT, which also indicated that the occurrence of EMT was accompanied by radioresistance in NSCLC cells.

DSBs are the most crucial lesions of radiation-induced DNA damage.^41^ The rapid phosphorylation of H2AX at Ser139 to produce γ-H2AX is a sensitive and reliable indicator of the cellular response to DSBs.^33^ In the present study, we found that the protein levels of γ-H2AX were increased 4 h after IR compared with non-irradiated cells. Moreover, the γ-H2AX levels were significantly lower in PC9-miR-410 and SPC-A1-miR-410 cells 4 h after radiation, whereas the opposite results were observed in A549-Inh and H1299-Inh cells. The data suggested that miR-410-increased radioresistance might be associated with enhanced DSB repair in NSCLC cells. However, whether miR-410 directly regulates genes involved in the major pathways of DNA damage repair (NHEJ and HR) or in the DNA damage response (DDR)-associated processes, including the cell cycle and apoptosis, still need to be further explored.

A single miRNA is able to target multiple genes by binding to the 3′-untranslated regions (3′-UTRs), which leads to the degradation of mRNA or the inhibition of mRNA translation.^14^ In our previous studies, we revealed that miR-410 promoted NSCLC tumorigenesis, development, stemness, and metastasis by targeting SLC34A2 and Gsk3β.^24,25^ miR-410 was also reported to enhance proliferation by targeting BDR7 in NSCLC.^26^ In the current study, three bioinformatic databases were used to predict the targets of miR-410. The results showed that PTEN was among the potential targets. Further KEGG analysis suggested that the potential targets of miR-410 were involved in cancer-related pathways, including Ras, PI3K/Akt, mTOR, and Wnt. Studies have demonstrated that the PI3K/Akt and mTOR pathways are closely associated with PTEN.^36^ Therefore, we hypothesized that PTEN might be a candidate target of miR-410. In the present study, the direct interaction between miR-410 and PTEN was confirmed by dual-luciferase reporter assays, and PTEN was found to be posttranscriptionally regulated by miR-410. These results indicated that PTEN was a direct target of miR-410 in NSCLC cells. Similarly, in a recent study, PTEN was also reported as the target of miR-410, and human umbilical cord mesenchymal stem cell-derived extracellular vesicles increased the growth of lung adenocarcinoma (LUAD) cells by transferring miR-410 to cells and subsequently decreasing the protein level of PTEN.^42^ However, the study mainly focused on exploring the effects and mechanism of hUCMSCs on LUAD growth without addressing the functions of miR-410 in EMT, radioresistance or the PI3K/mTOR pathway in lung cancer.

PTEN is a well-characterized negative regulator of the PI3K/Akt signaling pathway.^36^ Loss of PTEN in NSCLC drives the hyperactivation of PI3K/Akt and downstream mTOR, thus regulating multiple cellular functions.^43^ Studies also found that PTEN could mediate NSCLC EMT and radioresistance, respectively.^34,44^ Furthermore, in NSCLC, growing evidence proved that the activation of the PI3K/Akt/mTOR pathway could induce EMT,^33^ and played important roles in radioresistance as the downstream of the EGFR pathway.^38^ Dual inhibition of PI3K/Akt and mTOR enhanced the radioresponse by regulating the DDR process in various cancers, including NSCLC.^38,45^ Therefore, we hypothesized that miR-410 exerts its biological effects on EMT and radioresistance by activating the PI3K/mTOR pathway. Indeed, we found that overexpression of miR-410 in NSCLC cells robustly increased the levels of phosphorylated Akt, mTOR, P70S6K, and 4E-BP1. Conversely, knockdown of miR-410 remarkably decreased the levels of these markers. Our results further showed that treatment with a specific PI3K, Akt, or mTOR inhibitor drastically inhibited miR-410-induced EMT and radioresistance in PC9-miR-410 and SPC-A1-miR-410 cells. In addition, restoration of PTEN expression in PC9-miR-410 and SPC-A1-miR-410 cells significantly decreased miR-410-induced EMT and radioresistance, whereas transfecting siPTEN in A549-Inh and H1299-Inh cells resulted in the opposite effects. Consequently, our data demonstrated that PTEN was essential for miR-410-induced activation of the PI3K/mTOR pathway, which resulted in promoting both EMT and radioresistance in NSCLC cells (Fig. 7f). These results also indicated that PTEN played an important role in the biological functions induced by miR-410. Recently, Zhang et al.^46^ reported that miR-410-3p exerted oncogenic functions in prostate cancer via the PTEN/AKT/mTOR pathway. Yang et al.^47^ showed that loss of lncRNA OIP5-AS1 induced miR-410 accumulation and regulated its target KLF10/PTEN/AKT-mediated cellular behaviors in multiple myeloma cells. These data also showed the association between miR-410 and the PTEN/Akt/mTOR axis in tumors.

Studies showed that the expression of E-cadherin increased the radiosensitivity of breast cancer cells.^48^ Moreover, miR-205 inhibited DNA damage repair and acted as a radiosensitizer by targeting ZEB1 in breast cancer cells,^49^ whereas the promotion of DDR by ZEB1 might be the mechanism underlying the correlation between EMT and radioresistance.^50^ Hence, the roles of EMT-related markers and transcription factors in the radioresponse suggested that EMT might be one of the causes of radioresistance in cancers. Furthermore, a study on the tumor microenvironment of NSCLC demonstrated that the activation of the IGF1Rβ/PI3K/AKT pathway by hypoxia could induce EMT, which might subsequently enhance radioresistance.^51^ Consequently, we inferred that in NSCLC cells, radioresistance with enhanced DNA damage repair might be promoted by the miR-410-induced EMT process. However, to clearly understand the correlation between miR-410-induced EMT and radioresistance, a separate and effective tracing system is needed to monitor the EMT process because the EMT phenotype is reversible and transient. For instance, Fischer and Zheng et al.^52,53^ established effective tracking systems to monitor the EMT process in mice, thus clarifying the role of EMT in metastasis and chemoresistance. This issue will be explored in the future.

Further investigations in vivo showed that the levels of mesenchymal markers were remarkably increased, whereas the levels of E-cadherin and PTEN were significantly decreased, accompanied by the elevated expression of phosphorylated PI3K/mTOR pathway markers in SPC-A1-miR-410 tumors. The opposite observations were showed in A549-Inh tumors. Moreover, SPC-A1-miR-410 tumors were considerably more radioresistant, as demonstrated by a shorter growth delay and a reduction in γ-H2AX levels. Thus, these data suggested that miR-410 overexpression could enhance the EMT process and radioresistance in vivo, which was associated with the PTEN/PI3K/mTOR axis. Likewise, other miRNAs (e.g., miR-200c^17^) have been observed to regulate the radioresponse in lung cancer subcutaneous tumor models. Limitations still exist by using pathway inhibitors as radiosensitizers in NSCLC.^38,54^ Thus, our results suggest that miR-410 may serve as a potential target to improve the radioresponse of NSCLC.

In our previous studies, the upregulation of miR-410 was observed in NSCLC specimens.^20,21^ The results confirmed the role of miR-410 as an oncogene in NSCLC. Consistent with these findings, high miR-410 expression tended to be associated with poorer overall survival in lung adenocarcinoma patients in the current study, which further indicated the oncogenic role of miR-410. Moreover, mesenchymal and EMT phenotypes and high vimentin expression were more likely to be found in NSCLC patients with high miR-410 expression, and the protein levels of PTEN were positively associated with E-cadherin levels in TCGA NSCLC specimens. These results preliminarily suggested a positive correlation between miR-410 and EMT. However, except for the positive correlation between negative E-cadherin expression and low differentiation in 62 NSCLC samples, the expression of Vimentin and Slug showed no significant differences with the pathology characteristics. This might be owing to the limited number of samples. On the basis of the association between EMT and radioresistance, these data also preliminarily indicate that miR-410 might be a potential target of radioresponse in NSCLC. However, it needs to be explored further in post-RT samples.

In summary, we revealed that miR-410 induced both NSCLC EMT and radioresistance by targeting the PTEN/PI3K/mTOR axis in vitro and in vivo, and the promotion of radioresistance was associated with enhanced DNA damage repair. Moreover, the data also suggested that miR-410-induced EMT might significantly contribute to the enhanced radioresistance, which might be a novel mechanism of radioresistance in NSCLC. Further investigation in NSCLC specimens also demonstrated that miR-410 was positively correlated with EMT. Therefore, our results indicate that miR-410 may serve as a potential biomarker or therapeutic target in NSCLC RT, which might increase the treatment efficiency while inhibiting EMT-related malignancy.

## Materials and methods

### Cell culture, lentiviruses, and stable cell lines

The NSCLC cell lines A549, H1299, PC9, and SPC-A1, the human embryonic kidney cell line 293T (293T) and the normal lung epithelial cell line HBE were purchased from American Type Culture Collection (Manassas, VA, USA). A549, H1299, PC9, and SPC-A1 cells were maintained in RPMI 1640 (Invitrogen, Carlsbad, CA, USA); 293T and HBE cells were cultured in Dulbecco’s modified Eagle’s medium (Invitrogen) supplemented with 10% fetal bovine serum (Invitrogen) at 37 °C in 5% CO_2_.

Recombinant lentiviruses expressing miR-410 or the scrambled control were constructed and packaged by HanBio (Shanghai, China). The stable miR-410-overexpressing and control SPC-A1 and PC9 cell lines were established by lentivirus infection and puromycin (Merck Millipore, Burlington, MA, USA) selection as previously described.^25^ The expression efficiencies of miR-410 were quantified by quantitative real-time PCR (qRT-PCR). The miR-410-knockdown stable A549 and H1299 cell lines (A549-Inh and H1299-Inh) were established and confirmed previously.^25^

### Tissue specimens

A total of 62 clinical NSCLC specimens used in this study were obtained from the Department of Thoracic Surgery from patients histopathologically and clinically diagnosed at West China Hospital by procedures approved by the Medical Ethical Committee of West China Hospital, Sichuan University (Chengdu, China). Clinical information of the 62 NSCLC cases is presented in Supplemental Table S4.

### Quantitative real-time PCR

qRT-PCR was performed as previously described.^25^ Total RNA was extracted using TRIzol Reagent (Invitrogen) and was reverse-transcribed with the PrimeScript RT reagent Kit (Takara, Dalian, China). miR-410 (MIMAT0002171) stem-loop primers and U6 primers were commercially synthesized (RiboBio Co., Ltd, Guangzhou, China). The primers for qRT-PCR were as follows: PTEN (NM_000314) forward, 5′-TGTGGTCTGCCAGCTAAAGG-3′; PTEN reverse, 5′-CGGCTGAGGGAACTCAAAGT-3′. qRT-PCR was performed with the SYBR Green Real-Time PCR Master Mix Kit protocol (Bio-Rad, Hercules, CA, USA) on a CFX96 Real-Time System (Bio-Rad) to quantify the miR-410 or PTEN mRNA level using U6 or β-actin as an internal control, respectively. The relative expression of miR-410 and PTEN was calculated by the 2^−ΔΔCt^ method.

### Western blotting

Western blotting was performed as described previously.^25^ Primary antibodies against β-actin, E-cadherin, N-cadherin, γ-H2AX (Ser139), PTEN, PI3K, Akt, p-Akt (Ser473), mTOR, p-mTOR (Ser2448), P70S6K, p-P70S6K (Thr389), 4E-BP1, P-4E-BP1 (Ser65) (Cell Signaling Technology, Danvers, MA, USA), Slug (Santa Cruz Biotechnology, Dallas, TX, USA), and Vimentin (Zen BioScience, Chengdu, China) were used.

### Immunofluorescence analysis

Cells were plated onto coverslips in six-well dishes and cultured overnight, fixed with 4% paraformaldehyde in PBS, permeabilized with 0.5% Triton X-100 and blocked with 1% bovine serum albumin in PBS. For E-cadherin and Vimentin staining, cells were incubated with rabbit anti-E-cadherin antibody (Cell Signaling Technology) and mouse anti-Vimentin antibody (Zen BioScience) overnight at 4 °C, followed by detection with anti-rabbit-Alexa Fluor 647 conjugate (Cell Signaling Technology) or anti-mouse-TRITC (ZSGB-BIO, Beijing, China). For γ-H2AX staining, after culturing overnight, cells were irradiated with a dose of 4 Gy and incubated for 4 h. Cells were then fixed, permeabilized, blocked and incubated with rabbit anti-γ-H2AX (Cell Signaling Technology), followed by incubation with anti-rabbit-Alexa Fluor 647 conjugate (Cell Signaling Technology). For nuclear detection, cells were co-stained with 4′,6-diamidino-2-phenylindole (Sigma-Aldrich, St. Louis, MO, USA). Immunofluorescence images were acquired on a Zeiss Imager Z2 microscope (Carl Zeiss, Oberkochen, Germany).

### Clonogenic survival assay

The clonogenic survival assay was performed to evaluate the radioresponse of cells. Cells were seeded in triplicate in six-well dishes and allowed to stabilize overnight. The following day, cells were irradiated to a dose of 0, 2, 4, 6, or 8 Gy and incubated for 12 days to allow macroscopic colony formation. Colonies were then fixed with methanol and stained with 0.5% crystal violet. The number of colonies was counted with a cutoff of 50 viable cells per colony. Survival fraction was calculated relative to that of unirradiated cells (survival fraction = (plating efficiency of treated cells)/ (plating efficiency of control cells), where plating efficiency = (number of colonies formed by treated cells)/(number of colonies formed by untreated cells)).

### Plasmid construction, siRNA, and transfection

The wild-type and mutant 3′-UTR sequences of PTEN were cloned into the pmirGLO vector (Promega, Madison, WI, USA) respectively, and validated by sequencing. The PTEN expression plasmid was constructed by cloning PTEN cDNA (NM_000314) into the pVax vector. Three small interfering RNAs targeting human PTEN mRNA (named siPTEN) were designed for the RNA interference study (Supplemental Table S5). Lipofectamine 3000 (Invitrogen) was used for transfection according to the manufacturer’s instructions.

### Dual-luciferase assay

To validate the direct interaction between miR-410 and the 3′-UTR of PTEN, 293T cells were seeded in 96-well plates the day before transfection and then co-transfected with ~100 ng of wild-type or mutant PTEN 3′-UTR and with miR-410 mimics (50 nm) or inhibitors (100 nm) or negative control (50 nm) (RiboBio Co. Ltd). Luciferase activity was detected 24 h after transfection and assayed by using the Dual-Glo Luciferase Reporter System (Promega). All experiments were performed in triplicate. Firefly luciferase activity was normalized to the internal Renilla luciferase activity.

### Xenografted tumor model in vivo

To evaluate the effects of miR-410 on EMT and radioresistance in vivo, a nude mouse xenograft model was established. Five-week-old male athymic BALB/c nude mice (BEIJING HFK BIOSCIENCE CO. LTD, Beijing, China) were maintained in a specific pathogen-free (SPF) environment. SPC-A1-miR-410 or SPC-A1-miR-NC cells (5 × 10^6^) were injected subcutaneously into the right flanks of mice. One tumor per mouse was inoculated. Tumor volume (in mm^3^) was determined by caliper measurements performed twice a week and calculated by using the modified ellipse formula (volume = *π*/6 × length × widths^2^). When the xenograft tumor volumes reached ~200 mm^3^, mice were randomly assigned to mock RT or weekly 10 Gy dose of RT for 3 weeks.^55^ The tumor growth delay time (in days) was calculated by subtracting the average tumor volume quadrupling time for irradiated tumors from the average tumor volume quadrupling time for non-irradiated tumors.^56^ The tumor volume quadrupling time was calculated for each individual animal and then averaged for each group (*n* = 7/group). When the volumes of tumors reached four times the original volume, the mice were killed, and their tumors were harvested for immunohistochemical and western blotting analyses. All experiments involving mice were admitted and performed according to the requirements of the Institutional Animal Care and Use Committee of West China Hospital, Sichuan University (Chengdu, China).

### Immunohistochemical analysis

The tissues of xenograft tumors or clinical NSCLC samples were fixed in 4% paraformaldehyde and routinely dehydrated. Paraffin-embedded tissue samples were cut into 4 μm sections and deparaffinized, rehydrated, and then incubated with primary antibodies against E-cadherin, PTEN (Cell Signaling Technology), Vimentin (Zen BioScience) or Slug (Santa Cruz Biotechnology), respectively. Immunostaining was developed with 3–3’diaminobenzidine tetrahydrochloride (DAB) and co-stained with hematoxylin. Immunostaining images were acquired with a Zeiss AX10 Imager A2 microscope (Carl Zeiss).

The results of IHC were scored independently by two observers in terms of both the intensity and the extent of staining. The staining intensity was graded as 0, negative staining; 1, weak staining; 2, moderate staining; and 3, strong staining. The proportion of positively stained cells per specimen was determined as follows: 0 for no positively stained cells; 1 for <10%; 2 for 10–50%; and 3 for >50% of the examined cells. The histological score (H-score) was calculated by the proportion score × intensity score. An H-score ≥4 was considered high expression, and ≤3 was considered low expression. A final total score was graded as negative (−), weak (+), moderate (++), or strong (+++).

For the EMT phenotype assessment, the epithelial phenotype was defined as high E-cadherin expression and low expression of all mesenchymal phenotype biomarkers, whereas the mesenchymal phenotype was identified by high expression of one of the mesenchymal phenotype biomarkers together with low E-cadherin expression; the epithelial-to-mesenchymal phenotype was defined as high expression of both E-cadherin and mesenchymal phenotype biomarkers, whereas the remaining were not specified.^57^

### Analysis of NSCLC data in PROGmiRV2 or cBioPortal for TCGA database

TCGA is available from the website of the Cancer Genomics Browser of the University of California, Santa Cruz (https://genome-cancer.ucsc.edu/). The PROGmiRV2 and cBioPortal for Cancer Genomics are open-access downloaded biodatabases,^58,59^ providing visualization and analysis tools for large-scale cancer genomics datasets. Both portals collected records from the TCGA database. In total, 542 lung adenocarcinoma cases and 416 lung squamous cell carcinoma cases were performed by PROGmiRV2 to preliminarily analyze the correlation between the expression of miR-410 and overall survival. A total of 360 lung adenocarcinoma cases and 325 lung squamous cell carcinoma cases were performed by cBioPortal to preliminarily analyze the correlation between the protein expression of PTEN and EMT markers.

### Statistical analysis

Data were analyzed with GraphPad Prism 6 or SPSS 22.0 software. Significant differences between two groups were analyzed using two-tailed Student’s *t* test. The correlation between miR-410 expression and EMT phenotype, EMT marker expression or PTEN expression was analyzed by *χ*^2^ test. All values were presented as the mean ± SD. *P* < 0.05 (^*^/^▲^/^▼^), *P* < 0.01 (^**^/^▲▲^/^▼▼^), and *P* < 0.001 (^***^/^▲▲▲^/^▼▼▼^) were considered statistically significant.

## Supplementary information

Revised Supplementary Materials-Clean version
