# Supplementary material for: miR-410 induces both epithelial–mesenchymal transition and radioresistance through activation of the PI3K/mTOR pathway in non-small cell lung cancer
Source: Signal Transduct Target Ther. 2020 Jun 12;5:85. doi: 10.1038/s41392-020-0182-2 (PMC7290026; doi:10.1038/s41392-020-0182-2)
Supplement: Supplementary file 1 — Revised Supplementary Materials-Clean version [file 41392_2020_182_MOESM1_ESM.docx]

Supplementary Materials for

**miR-410 induces both epithelial-mesenchymal transition and radioresistance through activation of the PI3K/mTOR pathway in non-small cell lung cancer**

Yue Yuan^1^, Hu Liao^2^, Qiang Pu^2^, Xixian Ke^1^, Xueting Hu^1^, Yongfang Ma^1^, Xinmei Luo^1^, Qianqian Jiang^1^, Yi Gong^1^, Min Wu^3^,Lunxu Liu^2*^ and Wen Zhu^1*^

*^1^State Key Laboratory of Biotherapy and Cancer Center, ^2^Department of Thoracic Surgery, West China Hospital, Sichuan Universityand Collaborative Innovation Center of Biotherapy, Chengdu, Sichuan, China, ^3^Department of Biomedical Sciences, School of Medicine & Health Sciences, University of North Dakota, Grand Forks, ND 58203, USA*

**^*^ Corresponding author:** Wen Zhu, Ph.D. Prof.

**Mailing address:**State Key Laboratory of Biotherapy and Cancer Center, West China Hospital, Sichuan Universityand Collaborative Innovation Center of Biotherapy, No.1, Keyuan 4th Road, Gaopeng Street, High Technological Development Zone, Chengdu 610041, Sichuan Province, People’s Republic of China.**Tel:** +86-28-85164041, **Fax:** +86-28-85164041

**^*^Co-Corresponding author:**Lunxu Liu, M.D. Prof.

**Mailing address:** Department of Thoracic Surgery, West China Hospital, Sichuan University, Chengdu 610041, Sichuan Province, People’s Republic of China.**Tel:** +86-28-85422494, **Fax:** +86-28-85422494

**Correspondence to:**zhuwen@scu.edu.cn, lunxu_liu@aliyun.com

**This file includes:**

Figures S1 to S4

Tables S1 to S5


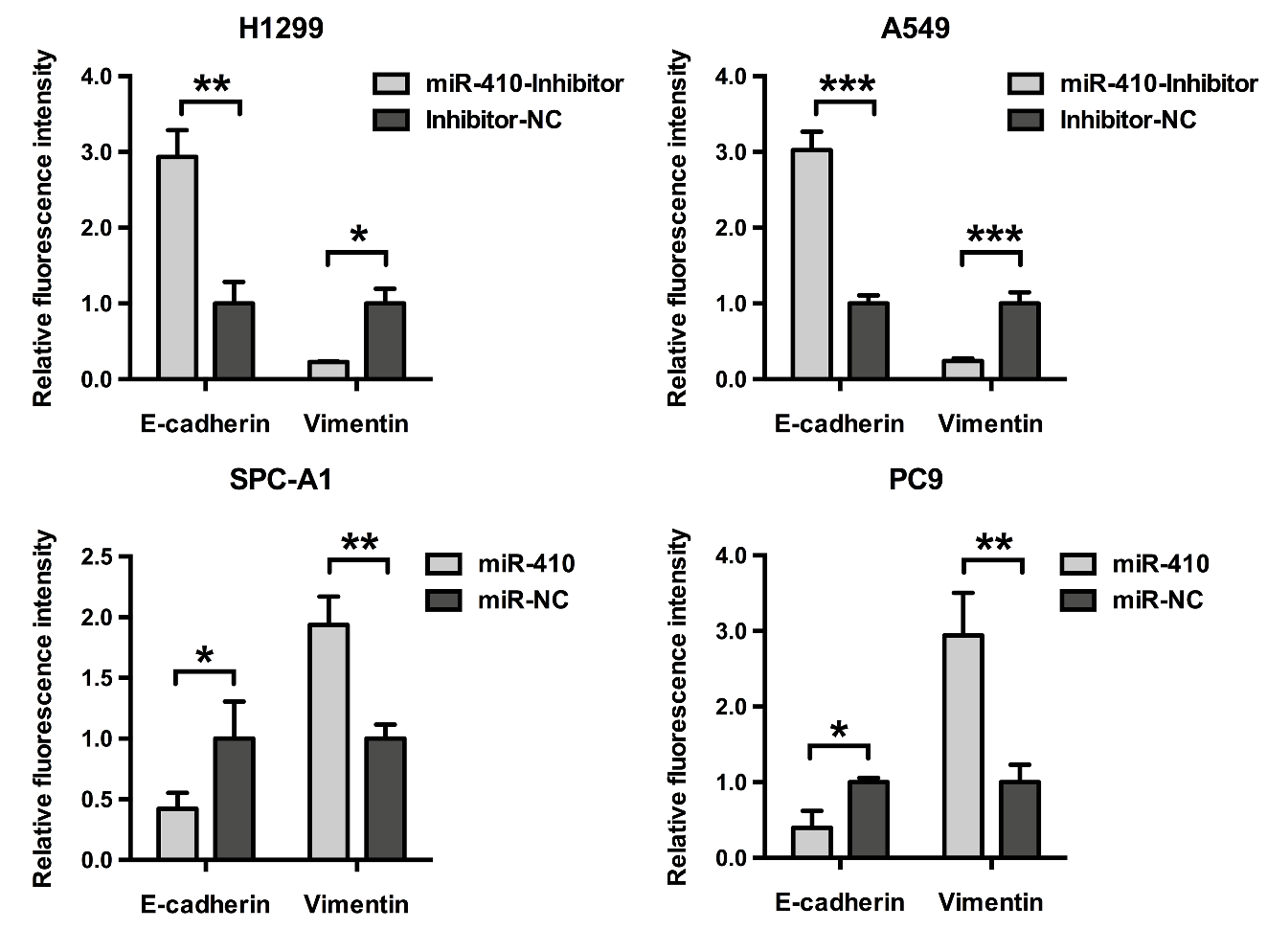


**FigureS1.** Relative fluorescence intensities of EMT markers (E-cadherin and Vimentin) in indicated cells.


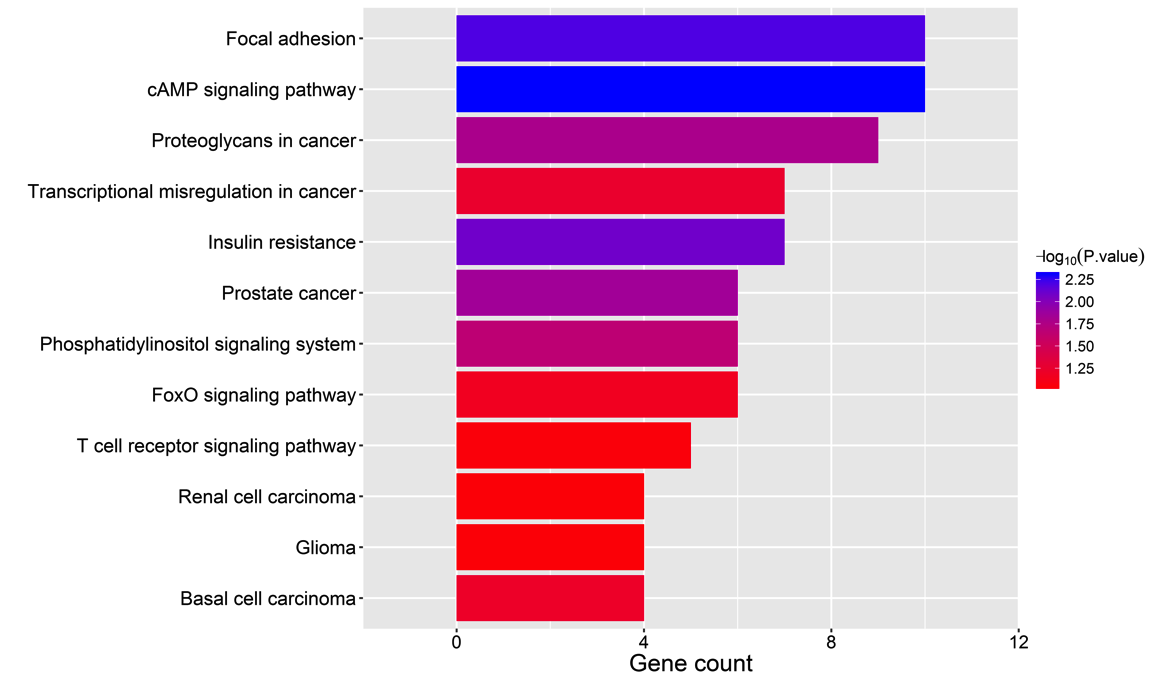


**FigureS2.**The KEGG analysis of miR-410 potential targets by DAVID bioinformatics resources. The potential target genes of miR-410 predicted by all the three algorithms (n=289) were involved incancer-related pathways.


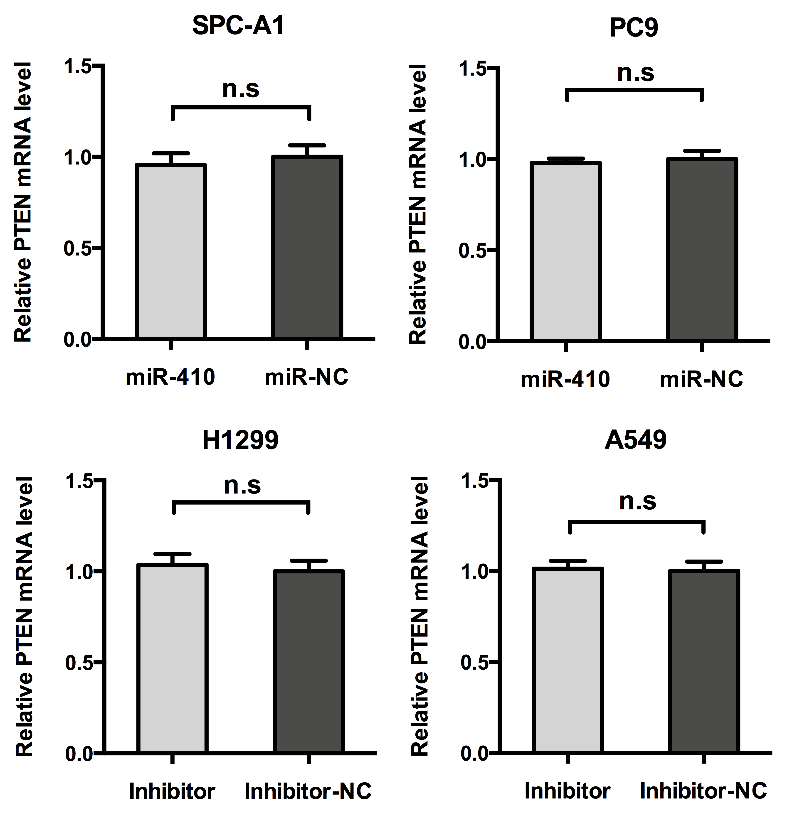


**FigureS3.** The mRNA levels of PTEN in indicated NSCLC cells.


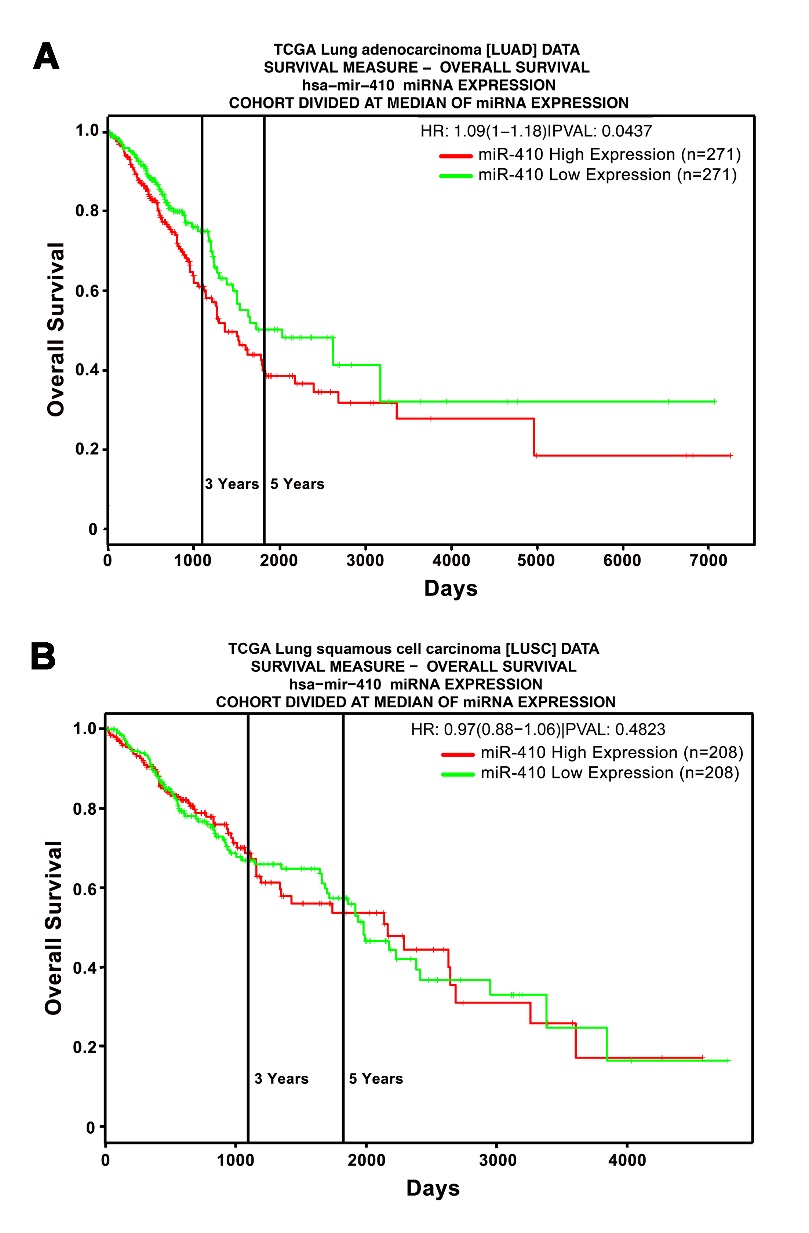


**FigureS4.** The correlations between miR-410 expression and overall survival in NSCLC patients from the TCGA database.(A) The correlations between miR-410 expression and overall survival in 542 LUAD patients from the TCGA database (TCGA_LUAD) by PROGmiRV2. (B) The correlation between miR-410 expression and overall survival of 416 LUAD patients from the TCGA database (TCGA_LUSC) by PROGmiRV2.

**TableS1.** Tumor growth delay (in days) in radiotherapy modelsof SPC-A1-miR-410 xenograft tumors

| Groups | T4V0(days) | TGD(days) |
| --- | --- | --- |
| miR-410 | 10.14 |  |
| miR-NC | 15.14 |  |
| miR-410+RT | 16.71 | 6.57 |
| miR-NC+RT | 26.28 | 11.14 |

T4V0: the number of days for tumor volume reached four times the original volume; TGD: tumor growth delay, which was defined in days as the tumor volume quadrupling time of treated tumors compared with that of untreated tumors; RT: radiotherapy.

**Table S2.** Correlations between expression of miR-410 and EMT phenotype status in 62 cases of NSCLC patients

| Characteristics | | EMT phenotype | | | | | *P* |
| --- | --- | --- | --- | --- | --- | --- | --- |
|  |  | Number (%)  N=62 | Epithelial (%)  n=10(16.13) | Mesenchymal (%)  n=22 (35.48) | EMT (%)  n=15 (24.19) | Not specified (%)  n=15 (24.19) |  |
| miR-410 Expression | High | 31(50.00) | 3(4.84) | 15(24.19) | 10(16.13) | 3(4.84) | 0.009** |
|  | Low | 31(50.00) | 7(11.29) | 7(11.29) | 5(8.06) | 12 (19.35) |  |

**Table S3.** Correlations between E-cadherin, Slug, Vimentin expression and the baseline characteristics in 62 cases of NSCLC tissues

| Characteristics | | Number (%) | E-cadherin Expression | | *P* | Slug Expression | | *P* | Vimentin Expression | | *P* |
| --- | --- | --- | --- | --- | --- | --- | --- | --- | --- | --- | --- |
|  |  |  | + (%)  n=36 | -(%)  n= 26 |  | + (%)  n=41 | -(%)  n= 21 |  | + (%)  n=29 | -(%)  n= 33 |  |
| Age (years) | ＞65 | 18 | 9 | 9 | 0.410 | 15 | 3 | 0.067 | 11 | 7 | 0.148 |
|  | ≤65 | 44 | 27 | 17 |  | 26 | 18 |  | 18 | 26 |  |
| Gender | Male | 44 | 28 | 16 | 0.165 | 30 | 14 | 0.593 | 18 | 26 | 0.148 |
|  | Female | 18 | 8 | 10 |  | 11 | 7 |  | 11 | 7 |  |
| Pathology | Squamous | 22 | 12 | 10 | 0.677 | 15 | 7 | 0.800 | 7 | 15 | 0.080 |
|  | Adenocarcinoma | 40 | 24 | 16 |  | 26 | 14 |  | 22 | 18 |  |
| Clinical Stages | Ⅰ-Ⅱ | 36 | 22 | 14 | 0.567 | 26 | 10 | 0.233 | 17 | 19 | 0.934 |
|  | Ⅲ-Ⅳ | 26 | 14 | 12 |  | 15 | 11 |  | 12 | 14 |  |
| Differentiation | Low | 37 | 16 | 21 | 0.004** | 22 | 15 | 0.177 | 19 | 18 | 0.380 |
|  | Medium | 25 | 20 | 5 |  | 19 | 6 |  | 10 | 15 |  |
| Lymph Nodes Metastatic | Yes | 33 | 21 | 12 | 0.343 | 21 | 12 | 0.658 | 16 | 17 | 0.773 |
|  | No | 29 | 15 | 14 |  | 20 | 9 |  | 13 | 16 |  |
| miR-410 Expression | High | 31 | 16 | 15 | 0.303 | 21 | 10 | 0.788 | 21 | 10 | 0.0009*** |
|  | Low | 31 | 20 | 11 |  | 20 | 11 |  | 8 | 23 |  |

**Table S4**. Clinical information of the 62 NSCLC cases

| Characteristics | | Number (%) (N=62) |
| --- | --- | --- |
| Age (years) | ＞65 | 18 (29.03) |
|  | ≤65 | 44 (70.97) |
| Gender | Male | 44 (70.97) |
|  | Female | 18 (29.03) |
| Pathology | Squamous | 22 (35.48) |
|  | Adenocarcinoma | 40 (64.52) |
| Clinical Stages | Ⅰ~Ⅱ | 36 (58.06) |
|  | Ⅲ~Ⅳ | 26 (41.94) |
| Distant Metastatic | Yes | 3 (4.84) |
|  | No | 59 (95.16) |
| Differentiation | Low | 37 (59.68) |
|  | Medium | 25(40.32) |
| Lymph Nodes Metastatic | Yes | 33(53.23) |
|  | No | 29(46.77) |

**Table S5**. siRNAs of PTEN (siPTEN) designed in this study

| siPTEN | Sequence (5’-3’) |
| --- | --- |
| #1-sense  #1-antisense | CGGGAAGACAAGUUCAUGUTT |
|  | ACAUGAACUUGUCUUCCCGTT |
| #2-sense  #2-antisense | GCUACCUGUUAAAGAAUCATT |
|  | UGAUUCUUUAACAGGUAGCTT |
| #3-sense  #3-antisense | GGUGUAAUGAUAUGUGCAUTT |
|  | AUGCACAUAUCAUUACACCTT |
